# Agarose-Encapsulated Multifunctional Microspheres with High Drug Payload and Multimodal Imaging Capability for Arterial Embolization–Associated Hyperthermia Therapy

**DOI:** 10.3390/molecules31203626

**Published:** 2026-10-12

**Authors:** Yuqing Zhang, Shuo Huang, Quanming Kou, Yindong Xu, Yizhou Xu, Lei Chen, Xiangyue Wang, Changsheng Shi

**Affiliations:** 1 School of Automation, Hangzhou Dianzi University, Hangzhou 310018, China; 15854113545@163.com (S.H.); quanming0221@gmail.com (Q.K.); 251060136@hdu.edu.cn (X.W.); 2 Department of Interventional Vascular Surgery, The Third Affiliated Hospital of Wenzhou Medical University, Wenzhou 325200, China; xuyindong98@163.com (Y.X.); xuyizhou98@163.com (Y.X.); 3 Department of Clinical Laboratory, The Third Affiliated Hospital of Wenzhou Medical University, Wenzhou 325200, China; chenleijoyce@163.com

**Keywords:** TACE, hyperthermia, drug delivery, multimodule imaging, high drug load capacity

## Abstract

Hepatocellular carcinoma (HCC) represents a leading cause of cancer-related mortality worldwide, and transarterial chemoembolization (TACE) remains the standard first-line locoregional therapy for intermediate-stage HCC. Nevertheless, conventional TACE frequently yields suboptimal therapeutic efficacy in advanced or heterogeneous tumors, necessitating combined therapeutic strategies. Herein, we report a one-step emulsion fabrication strategy to develop agarose-encapsulated multifunctional microspheres (AEMMs) using low-cost, biocompatible agarose as the scaffold matrix. AEMMs co-encapsulate PSS for electrostatic drug binding, iodixanol as a dual-contrast agent for CT and DSA, and superparamagnetic Fe_3_O_4_ nanoparticles that serve as both MRI contrast agent and NIR photothermal converter. In vitro evaluation demonstrates that AEMMs achieve ultrahigh drug loading capacities of 89.87 ± 1.78 mg/mL for DOX or 90.60 ± 0.84 mg/mL for Irinotecan, which are comparable to commercial CalliSpheres^®^ and nearly twofold higher than DC Beads^®^. Photothermal evaluation confirms that AEMMs induce rapid temperature elevation above 50 °C within 3 min under 808 nm laser irradiation, with excellent thermal stability and reproducibility over repeated on–off cycles. Furthermore, AEMMs provide excellent complementary contrast for CT, DSA, and T_2_-weighted MRI, enabling real-time embolization monitoring and postoperative assessment. Blank AEMMs exhibit negligible cytotoxicity against HepG2 cells even at high concentrations, verifying favorable biocompatibility. Collectively, AEMMs integrate mechanical embolization, localized sustained chemotherapy, on-demand photothermal ablation, and multimodal imaging guidance into a single platform, offering a rational and promising integrated strategy to overcome the key limitations of conventional TACE for advanced HCC management.

## 1. Introduction

Primary hepatocellular carcinoma (HCC) is a highly aggressive malignancy with rising global incidence and mortality rates. Due to the lack of specific symptoms in the early stages, most patients are diagnosed at an intermediate or advanced stage, missing the optimal window for surgical treatment [1,2]. With the rapid development of minimally invasive interventional techniques, transarterial chemoembolization (TACE) has emerged as the first-line recommended therapy for unresectable intermediate to advanced liver cancer [3]. The therapeutic principle of TACE lies in performing super-selective catheterization to the tumor-feeding arteries, while simultaneously performing vascular embolization and local chemotherapy drug infusion, thereby achieving the dual effects of “starving” and “poisoning” the tumor [4]. The efficacy of this therapy highly depends on the selection and application of embolic materials. The traditional TACE procedure typically involves mixing iodized oil with chemotherapy drugs and injecting them [5,6]. Iodized oil has the dual functions of a drug carrier and an embolic agent [7]. By blocking the blood supply arteries to the tumor and allowing high concentrations of chemotherapy drugs to continuously act on the tumor tissue locally, it aims to directly or indirectly kill tumor cells, inhibit recurrence and metastasis. Compared with systemic chemotherapy, arterial administration can somewhat limit systemic drug diffusion [8], but there is still a risk of the drug leaking into the systemic circulation and damaging normal organs. In contrast, solid embolic agents such as embolic microspheres offer significant advantages, including precise tumor-site embolization, suppressed drug diffusion, and mitigation of the limitations associated with liquid embolic agents [9,10]. Among these, drug-eluting beads (DEBs) have become a pivotal platform in tumor interventional therapy [11]. By targeting the tumor’s feeding arteries, DEBs can physically block the blood vessels, cutting off the nutrient supply to the tumor [12], while gradually releasing chemotherapeutic or even targeted drugs, further maximizing the benefits of TACE.

Currently, drug-eluting embolic microspheres have been widely adopted in clinical chemotherapy embolization [13,14], but they still have significant deficiencies in imaging visibility and in vivo tracing (such as DC Beads^®^ from Biocompatibles UK; HepaSphere™ from Merit Medical Inc) [15,16,17,18]. Since these microspheres are not inherently visible under imaging, it is difficult to accurately determine their distribution and position within the blood vessels during embolization, which affects the assessment of treatment efficacy [19]. The key advantage of multimodal imaging embolic microspheres is that they allow real-time monitoring of microsphere injection and positioning using clinical imaging techniques such as computed tomography (CT), digital subtraction angiography (DSA), or magnetic resonance imaging (MRI), which enhances the precision of the procedure, helps avoid mis-embolization or under-embolization, and reduces damage to adjacent normal tissues [20]. Therefore, the development of efficient and precise embolic agents with imaging functions holds significant clinical importance for improving the long-term prognosis of patients.

Nevertheless, due to the rich collateral circulation in liver tissue and the strong angiogenic capability of the tumor itself, TACE alone often fails to achieve the desired therapeutic effect [21]. Accordingly, TACE is frequently combined with other therapeutic modalities in clinical practice, such as targeted therapy, radiotherapy, hyperthermia, radiofrequency ablation, and immunotherapy, to augment antitumor efficacy. Among them, tumor hyperthermia, as an important physical adjuvant treatment method, has attracted much attention in recent years [22]. Accumulating evidence [23,24,25,26] demonstrates that tumor tissues exhibit disordered microvascular architecture, insufficient blood perfusion, and poor heat dissipation capacity, resulting in significantly higher local temperatures than normal tissues upon heat stimulation.

When local temperature rises to 42–45 °C, the proteins and structures within cancer cells become damaged, ultimately result to apoptosis or necrosis. In contrast, normal tissues can effectively dissipate heat via intact blood circulation, thus avoiding thermal injury even under equivalent heat exposure. Studies have shown that combining radiotherapy and hyperthermia after TACE can enhance short-term efficacy in patients with intermediate to advanced liver cancer, improve survival rates, and reduce adverse reactions. The efficacy of combining hyperthermia with TACE is significant, and its safety is widely recognized. Hyperthermia not only increases cancer cells’ sensitivity to drugs but also improves blood flow and vascular permeability in the tumor, facilitating better penetration of chemotherapeutic agents [27]. Additionally, hyperthermia can enhance the body’s immune response and reduce the toxic side effects of chemotherapy and radiotherapy, earning it the designation of “green therapy” in the international medical community.

An ideal embolic microsphere should simultaneously achieve imaging, embolization, chemotherapy, and hyperthermia, employing multiple therapeutic mechanisms to deliver a comprehensive attack on tumors and optimize patient outcomes. In our previous work, we reported a type of multimodal imaging nano-assembled agarose microspheres (NAAMs) formed via host–guest assembly and surface-modified with sulfonic acid groups for drug binding. However, the preparation process of nano-assembled microspheres is complex and susceptible to external environmental fluctuations (e.g., temperature or pH variations, enzymatic degradation, shear forces, and ionic strength), which may cause the detachment of nanoscale spheres from the microsphere surface, potentially affecting both imaging and therapeutic efficacy.

**Figure 1:**
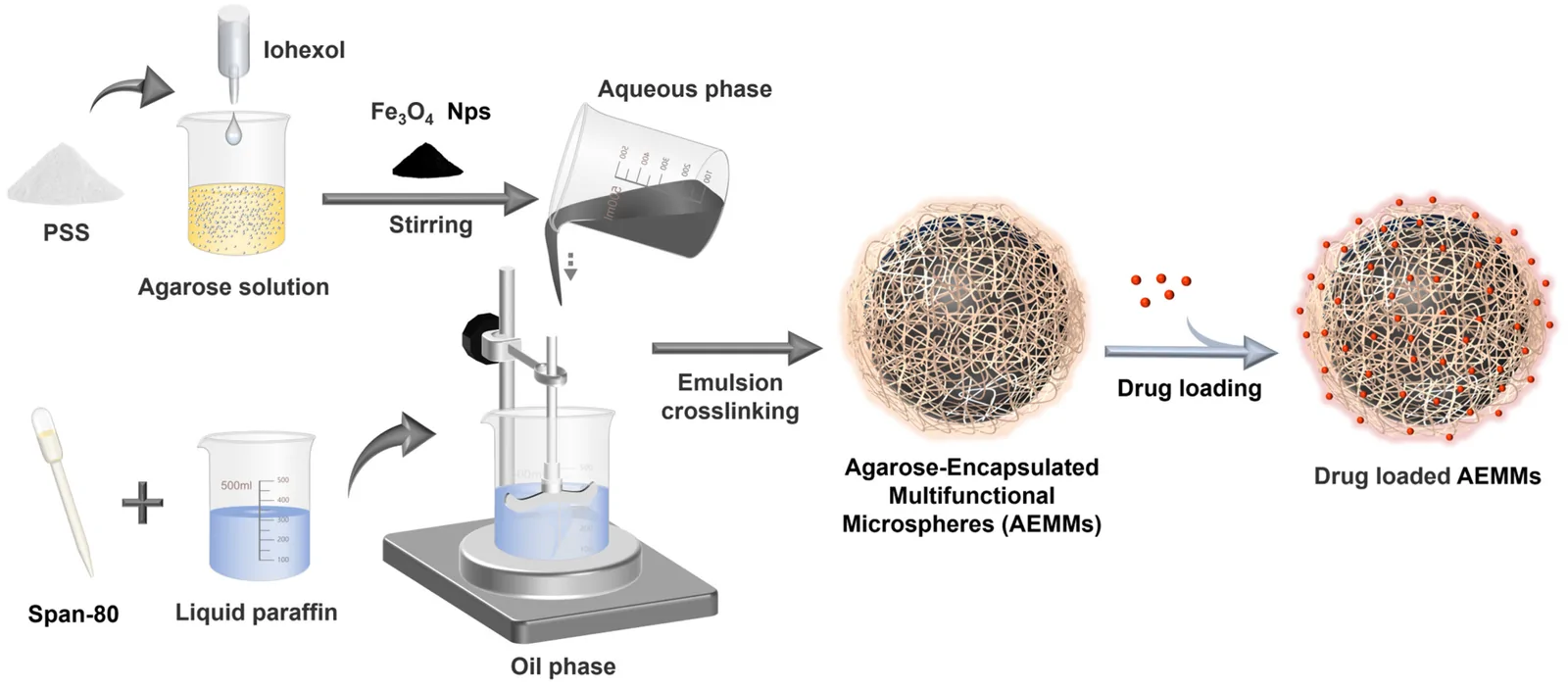
Schematic illustration of agarose-encapsulated multifunctional microspheres (AEMMs). The names and functional roles of key components: Iohexol (CT/DSA dual-mode contrast agent), superparamagnetic Fe_3_O_4_ Nps (MRI contrast agent and NIR photothermal conversion medium), poly(sodium-*p*-styrenesulfonate, PSS) (electrostatic drug-binding functional group), and Span 80 (emulsifier).

In this study, we innovatively developed a “one-step” emulsion method to fabricate Agarose-Encapsulated Multifunctional Microspheres (AEMMs), in which drug-binding functional groups, imaging agents, and photothermal conversion agents are simultaneously encapsulated within a single agarose microsphere (Figure 1).

This approach not only ensures multifunctionality but also effectively stabilizes the microsphere structure. The AEMMs offer the following advantages: (1) Multimodal imaging helps to improve the accuracy of embolization and monitoring. Fe_3_O_4_ nanoparticles are embedded in AEMMs to enhance the contrast of MRI, and iodohyl is used for CT and DSA imaging. Imaging information of different modes helps to monitor the distribution of microspheres in vivo and the location of embolization in real time during treatment. (2) Efficient drug loading and sustained-release: Experimental results show that 1 mL of AEMMs can load 89.87 ± 1.78 mg of doxorubicin hydrochloride (DOX) or 90.60 ± 0.84 mg of Irinotecan hydrochloride respectively, with the cumulative release closely matching the drug loading amount. The drug loading and release performance of AEMMs are comparable to the commercially available DC Beads^®^ and CalliSpheres^®^. (3) Combination of Photothermal Therapy (PTT) and Chemotherapy: The integration of PTT and chemotherapy enhances the sensitivity of cancer cells to drugs through thermal effects, improving overall therapeutic efficacy [28]. It may also induce tumor cell death and antigen release, potentially activate the immune system and further enhance the anti-tumor immune response. (4) Cost-effective manufacturing: Agarose, used as the main structural component of AEMMs, is a low-cost natural material that facilitates the clinical adoption of AEMMs. It also has a low risk of inducing immune or inflammatory responses and exhibits good thermal stability.

## 2. Result and Discussion

### 2.1. Characterization of AEMMs

**Figure 2:**
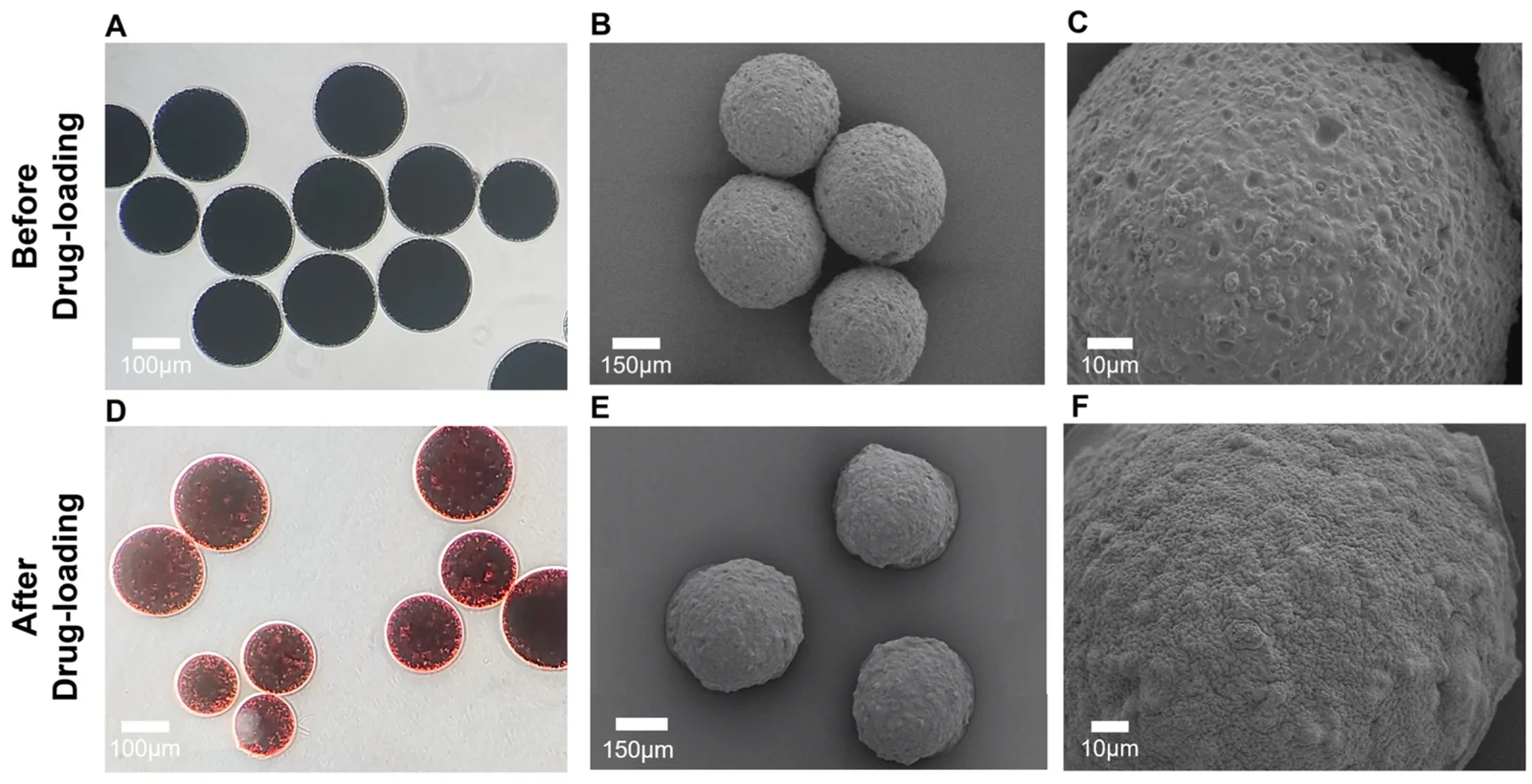
Characterization of AEMMs. Optical microscopy images of AEMMs before (**A**) and after (**D**) drug loading, with a scale bar of 100 μm. Representative low-resolution SEM images of AEMMs before (**B**) and after (**E**) drug loading, with a scale bar of 150 μm. High-resolution SEM images of AEMMs before (**C**) and after (**F**) drug loading, the scale bar is 10 μm.

First, the morphology of AEMMs before and after drug loading was characterized using optical microscopy and SEM. Figure 2A shows the optical microscopy image of AEMMs before drug loading. The average diameter is approximately 250 ± 15 μm, with a size range of 150–300 μm (Figure S1). Before loading, the microspheres appear black due to the presence of a large number of Fe_3_O_4_ Nps inside, while the outermost layer encapsulated in agarose is mostly transparent. After drug loading, as shown in Figure 2D, the microspheres display an orange-red color consistent with the loaded drug, DOX, which reflects the good drug loading performance of the microsphere. SEM images (Figure 2B,E and Figure S2) provide a detailed morphology of the AEMMs before and after drug loading. A magnified view in Figure 2C highlights the rough surface of AEMMs before drug loading, while after loading, a dense drug coating forms on the surface, maintaining the spherical shape. A comparison clearly shows that after drug loading, the drug molecules smoothen the microsphere surface, effectively filling the rough structure (Figure 2F). Notably, the particle size of AEMMs can be flexibly tuned in the range of 50–500 μm by modulating synthetic parameters. Specifically, higher stirring speed, higher Span 80 concentration, and lower aqueous-to-oil phase ratio generally yield smaller microspheres. This size tunability enables AEMMs to match the diameters of different target blood vessels in clinical embolization applications.

### 2.2. Evaluation of Drug Loading Capacity and Sustained-Release of AEMMs

**Figure 3:**
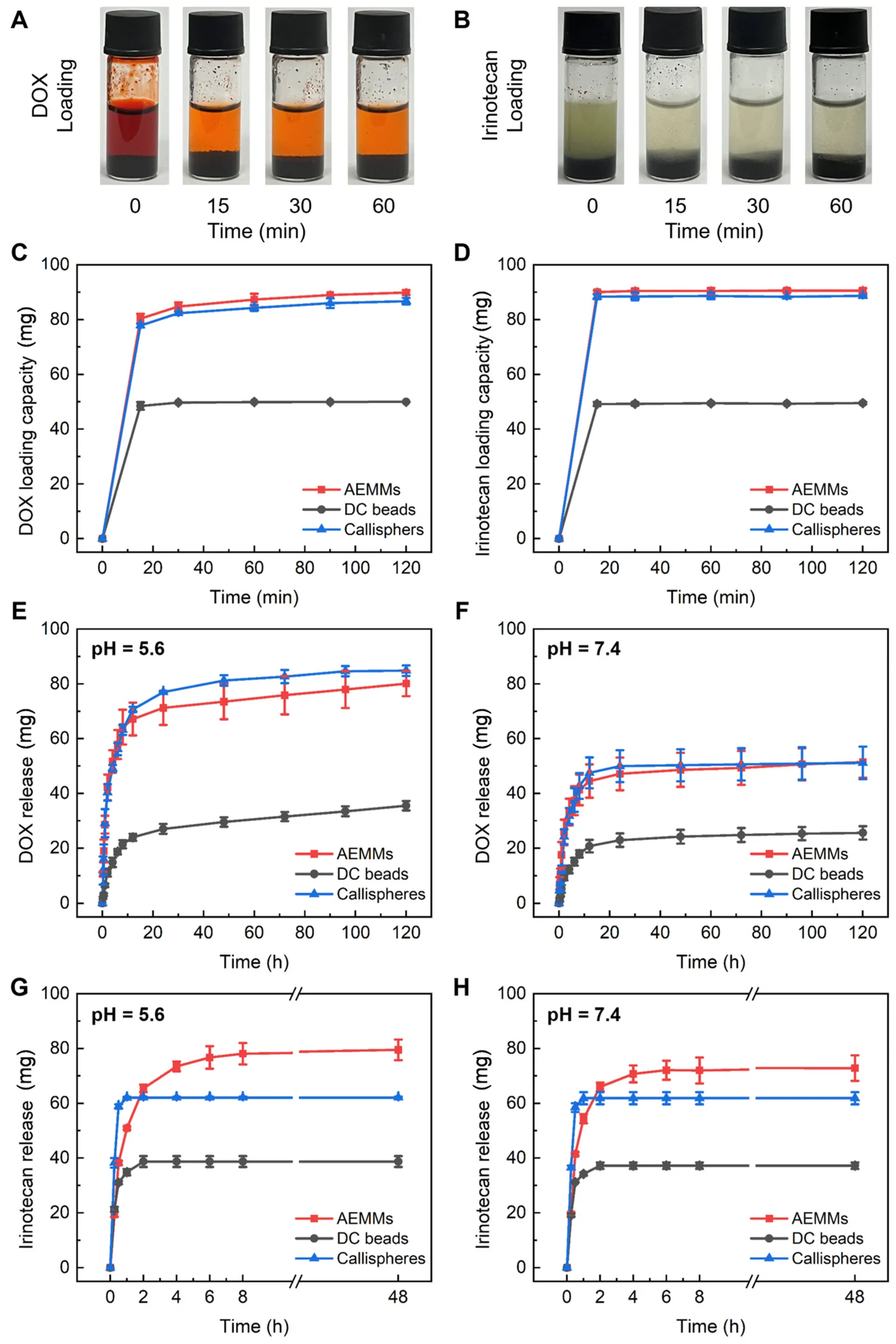
Drug loading and sustained-release performance. Photograph of suspensions of AEMMs loading DOX (**A**) and Irinotecan (**B**) in 0, 15, 30, 60 min. (**C**) The drug loading capacity of 1 mL microspheres (AEMMs, DC Beads, and Callispheres) for Doxorubicin (100 mg) and (**D**) Irinotecan (100 mg), error bars represent the standard deviation (*n* = 3). (**E**,**F**) DOX releases from AEMMs, DC Beads, and Callispheres in PBS (pH = 5.6 or pH = 7.4). (**G**,**H**) Irinotecan releases from AEMMs, DC Beads, and Callispheres in PBS (pH = 5.6 or pH = 7.4). Error bars represent the standard deviation (*n* = 3).

Doxorubicin (DOX) and Irinotecan are commonly used chemotherapeutic agents with broad anticancer activity, effective against various tumor cells (DOX is frequently used for liver cancer, breast cancer, leukemia, lymphoma, and soft tissue sarcoma, while Irinotecan is primarily used for colorectal cancer and other solid tumors). For different applications, AEMMs can efficiently load these drugs through covalent bonding accordingly. Figure 3A illustrates the DOX loading process in AEMMs. It shows that the color of the supernatant (orange-red from DOX) gradually lightens over time at 0, 15, 30, and 60 min, indicating a decrease in residual drug concentration. Similarly, Figure 3B depicts the Irinotecan loading process, where the initially turbid Irinotecan-sorbitol-lactic acid solution becomes clear as it is loaded onto the microspheres.

To evaluate the drug loading capacity and potential applications of AEMMs, we compared the performance with two commonly used clinical embolic microspheres: DC beads^®^ and Callispheres^®^. For consistency, we used 1 mL of each microsphere type for our comparisons, even though DC beads are typically sold in 2 mL packages. DOX has a primary absorption peak at 480 nm (±3 nm), and Irinotecan shows significant absorption at 254 nm and 369 nm. Drug loading was assessed using UV-vis absorbance spectra to measure the amount of DOX (Figure 3C) and Irinotecan (Figure 3D) loaded onto 1 mL of each microsphere type. Repeated experiments showed that, with the same amount of DOX added, AEMMs, DC beads, and Callispheres all quickly reached saturation within the first 30 min and then maintained stable loading. The drug loading capacities were 89.87 ± 1.78 mg/mL for AEMMs, 49.97 ± 0.16 mg/mL for DC beads, and 88.66 ± 0.86 mg/mL for Callispheres. Parallel experiments with Irinotecan showed similar results, with maximum loading achieved in the initial 30 min: AEMMs at 90.60 ± 0.84 mg/mL, DC beads at 49.52 ± 0.41 mg/mL, and Callispheres at 86.74 ± 1.18 mg/mL. AEMMs demonstrated excellent drug delivery capacity, achieving loading amounts comparable to Callispheres and nearly twice that of DC beads for the same volume. Considering clinical application dosages, AEMMs meet the required drug loading and efficiency while being extremely cost-effective. These results indicate that AEMMs exhibit superior drug loading performance for both drugs.

Next, we examined the drug cumulative release extent of AEMMs and compared them with commercially available drug-loaded microspheres. For DOX, the cumulative release results showed an initial burst release within the first 12 h for all three types of microspheres, followed by a gradual plateau, and then a steady increase. As shown in Figure 3E, in PBS at pH 5.6, the cumulative release of AEMMs, DC beads, and Callispheres over 120 h approached their maximum loading capacities, with values of 80.11 ± 4.60 mg, 35.54 ± 1.73 mg, and 84.84 ± 1.93 mg, respectively. The corresponding cumulative release extent were 90.0% for AEMMs, 71.1% for DC beads, and 97.8% for Callispheres, indicating sustained and stable release over time. In contrast, at pH 7.4, the cumulative release of AEMMs, DC beads, and Callispheres over 120 h was lower, with values of 51.43 ± 5.68 mg, 25.61 ± 2.42 mg, and 51.15 ± 5.92 mg, respectively, with cumulative release extent of 57.2% for AEMMs, 51.2% for DC beads, and 58.9% for Callispheres. This significant reduction in release at neutral pH demonstrates the strong pH-dependence of the drug release mechanism. In contrast, the weakly acidic microenvironment of tumor tissues is more conducive to the drug release from the microspheres.

Figure 3G,H shows the release performance of Irinotecan from the three types of microspheres. Unlike DC beads and Callispheres, which complete the release process within the first 2 h, AEMMs display the advantage of achieving a slower and more sustained drug release. This prolonged local drug concentration during treatment is of significant clinical importance for cancer therapy. In PBS with pH = 5.6, the cumulative release of Irinotecan over 48 h is 79.53 ± 3.77 mg for AEMMs, 38.73 ± 1.98 mg for DC beads, and 62.06 ± 0.34 mg for Callispheres. This indicates that, under the same conditions, AEMMs can release a greater amount of effective drug, thereby reducing drug waste, cutting costs, and achieving better therapeutic outcomes. In PBS with pH = 7.4, the cumulative release of Irinotecan over 48 h is 72.85 ± 4.64 mg for AEMMs, 37.22 ± 1.21 mg for DC beads, and 61.86 ± 2.25 mg for Callispheres. While DC beads and Callispheres show no obvious difference in release between neutral and acidic environments, AEMMs exhibit greater therapeutic potential in the acidic microenvironment typical of tumors.

### 2.3. Photothermal Properties of AEMMs

Research [29,30] indicates that combining TACE with hyperthermia can enhance the efficacy of treatment for intermediate and advanced liver cancer. When the temperature rises to between 42 °C and 45 °C, it not only inhibits the synthesis of DNA, RNA, and proteins in tumor cells but also affects the state and function of the cell membranes, increasing their permeability. Tumors typically have structurally abnormal angiogenic vessels with discontinuous endothelium and impaired blood perfusion, whereas normal tissues possess intact, well-organized blood circulation that effectively dissipates heat. Therefore, hyperthermia can raise the tumor temperature to cytotoxic levels while sparing surrounding normal tissues. Additionally, related studies have shown that hyperthermia can effectively alleviate post-TACE liver pain, improve the quality of life, reduce angiogenesis factors, and enhance immune regulation.

**Figure 4:**
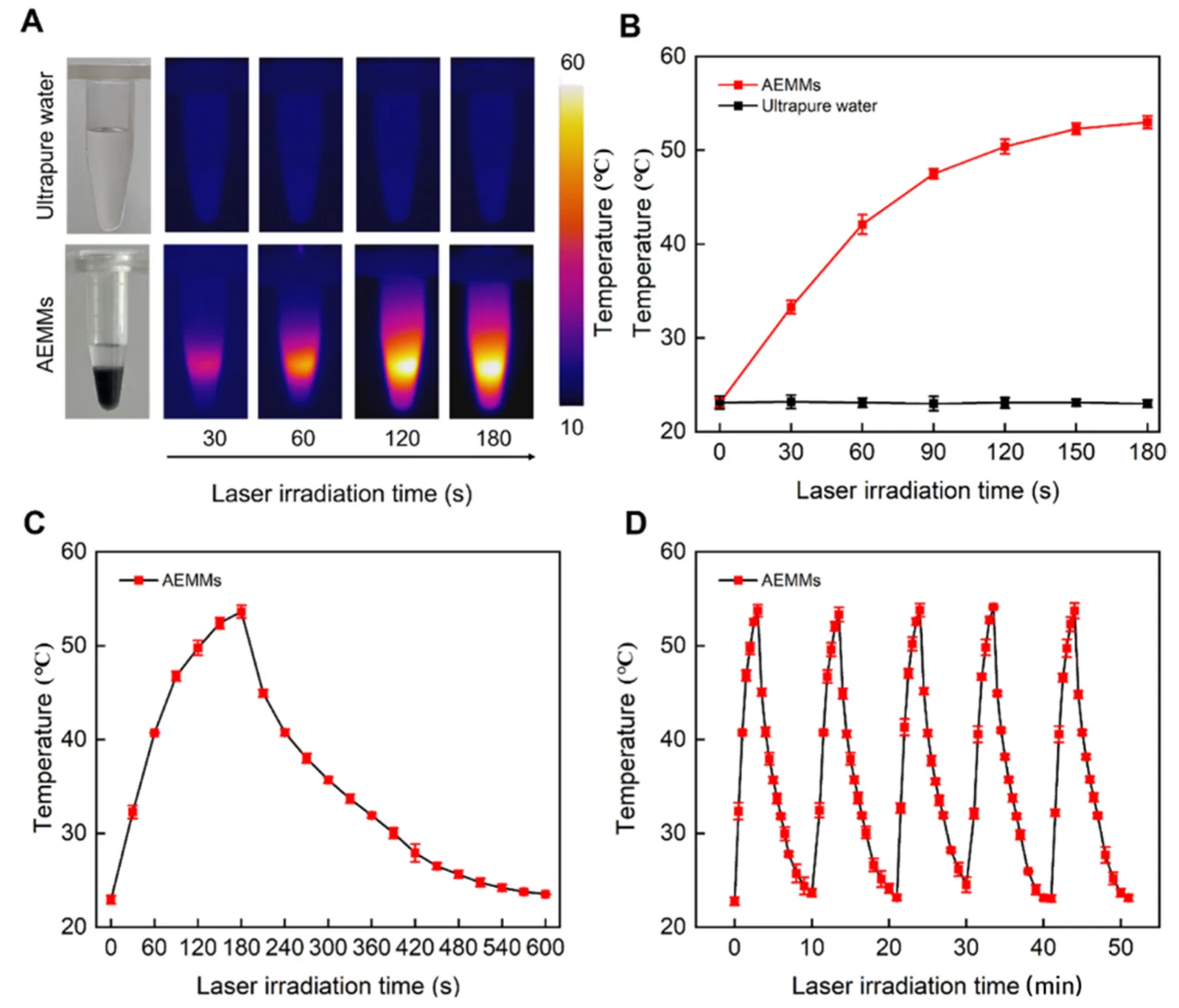
In vitro photothermal capacity evaluation. (**A**) Real-time photothermal images and (**B**) temperature curve of AEMMs and water irradiated by laser (808 nm, 0.33 W/cm, 180 s). (**C**) AEMMs photothermal capability test. (**D**) Photothermal stability test with five laser on/off cycles at 0.33 W/cm^2^.

We tested the photothermal capabilities of AEMMs using a 808 nm near-infrared laser as the light source. As shown in Figure 4A,B, the temperature of the AEMMs in the centrifuge tube increased significantly under laser irradiation, rising from 23.7 °C to 54.3 °C within 3 min, as monitored by the infrared thermal imaging camera. When tumor cells are exposed to temperatures above 42 °C for more than 5 min, they can be effectively destroyed. Under the same experimental conditions, the temperature increase in pure water was negligible, confirming that the Fe_3_O_4_ Nps within the AEMMs were responsible for the temperature rise. The results demonstrate that AEMMs can heat up rapidly and return to room temperature within 12 min after reaching 54.3 °C in 3 min (Figure 4C). These findings indicate that AEMMs exhibit excellent photothermal performance in the near-infrared range, with high photothermal conversion efficiency, meeting the expectations of the experiment. Additionally, we conducted tests to assess the photothermal stability of AEMMs (see Figure 4D). The AEMMs were irradiated with a 808 nm laser (0.33 W/cm^2^) for 300 s (LASER ON), followed by cooling to the initial temperature (LASER OFF), repeated over 5 LASER ON/OFF cycles. The results showed that the maximum temperature reached during each irradiation remained consistent, the cooling time from the maximum to the minimum temperature was unchanged, and the heating time to reach the maximum temperature also remained stable, confirming the excellent photothermal stability and repeatability of AEMMs. These properties make AEMMs an ideal carrier material for photothermal ablation of tumor cells, supporting repeated hyperthermia treatments. Clinically, NIR laser irradiation can be delivered via an optical fiber inserted through the working channel of the transarterial catheter during TACE, enabling localized photothermal ablation directly at the tumor embolization site. Alternatively, percutaneous NIR irradiation can be applied under imaging guidance post-embolization. The Fe_3_O_4_-mediated photothermal effect is confined to the microsphere-deposited tumor region, minimizing damage to surrounding normal liver tissue. Nevertheless, further studies are needed to evaluate the long-term effects and to explore optimal combinations of TACE, intra-arterial chemotherapy, and hyperthermia in terms of timing and dosage.

### 2.4. In Vitro Multimodal Imaging of AEMMs

In conventional TACE procedures, physicians mainly rely on experience and indirect imaging to determine the embolization location and dosage [31,32]. However, multimodal imaging embolization microspheres can display their accumulation in the target area through CT, X-ray, or MRI, allowing doctors to observe the distribution and extent of embolization in real-time. This enables more precise adjustments to dosage and positioning, helping to avoid mis embolization or insufficient embolization. As a result, damage to non-target tissues can be minimized, and critical surrounding tissues can be better protected, ultimately optimizing postoperative monitoring for patients.

The AEMMs embed significant amounts of Fe_3_O_4_ Nps and iohexol. Fe_3_O_4_ Nps, a superparamagnetic nanoparticle, serves as a contrast agent for magnetic resonance imaging (MRI) [33]. Due to its distinct behavior in magnetic fields compared to surrounding tissues, it enhances image contrast. Specifically, Fe_3_O_4_ Nps accelerates T_2_ relaxation times, generating higher signal intensity in images, which aids in clearer visualization of tissues and lesions [34]. Fe_3_O_4_ Nps can also function as a magnetic contrast agent in ultrasound imaging by altering the echo of ultrasound signals to improve image clarity. Iohexol, an iodine-based contrast agent, is commonly used in CT scans. Since iodine has a high absorption rate for X-rays, regions containing iodine appear more distinct in CT images [35]. By combining the strengths of these two materials, AEMMs can be utilized for MRI, DSA, and CT scanning, providing more comprehensive and precise imaging for detailed diagnostic results.

**Figure 5:**
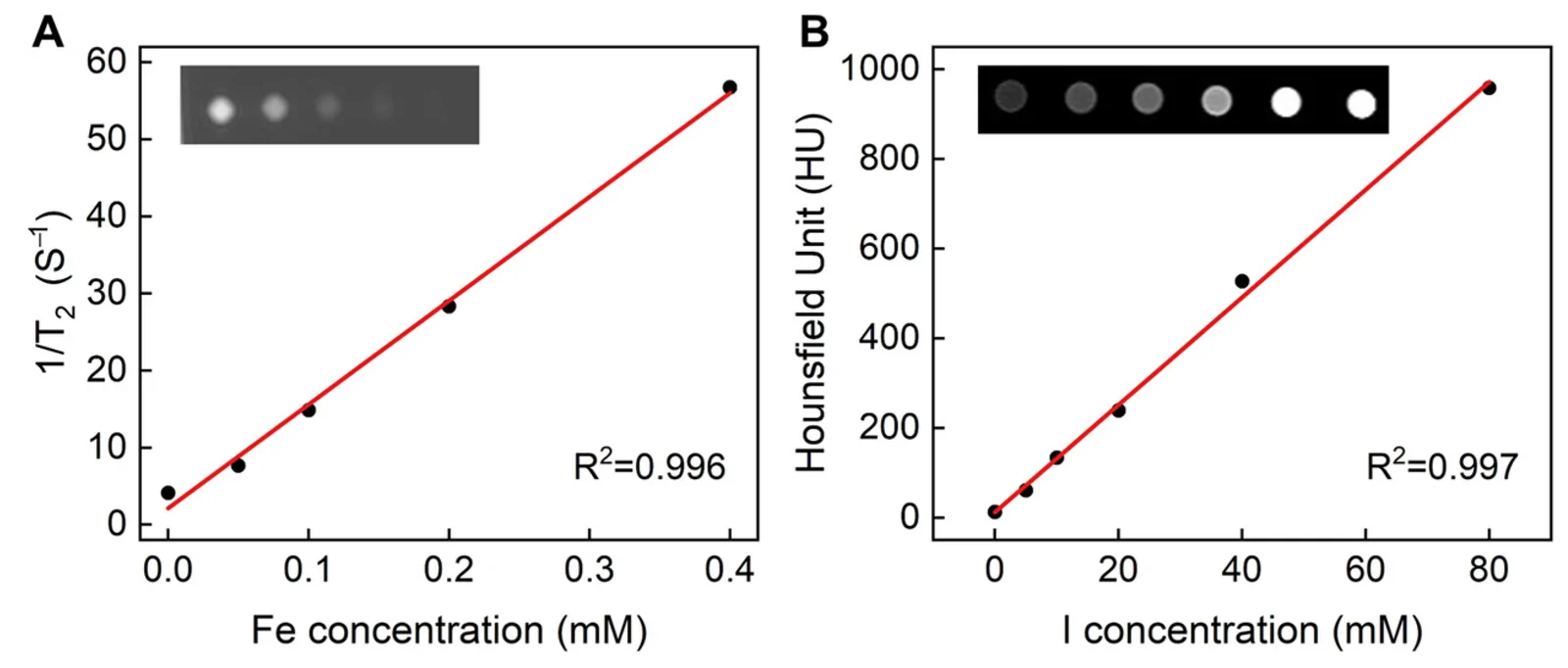
(**A**) Plot of R2 signal intensity versus agarose gel models concentrations in 1% agarose phantoms and (inset) MRI T_2_-weighted images at TE = 32 ms showing the expected contrast effects by the agarose gel models in phantom and (**B**) Concentration versus CT signal (Hounsfield units, HU) curve of the agarose gel models. Inset shows CT images of the agarose gel models comparing with 1% agarose phantoms.

Fe_3_O_4_-based materials are widely considered excellent candidates for T_2_-weighted MRI contrast agents. AEMMs demonstrate dose-dependent positive contrast enhancement, with signal intensity increasing as the iron concentration rises from 0 to 0.4 mM (Figure 5A). During MR imaging, the signal intensity of the agarose phantom embedded with 20 wt% magnetic clusters shows a strong linear correlation with concentration (R^2^ = 0.996). Figure 5B shows the DSA image of the AEMMs agarose gel model, where the X-ray attenuation intensity correlating with iodine content significantly increases. As the iodine concentration within the AEMMs rises from 0 to 80 mM, the brightness of the DSA virtual model image also increases, exhibiting a strong linear correlation (R^2^ = 0.997). This multimodal imaging reduces patient exposure to X-rays by allowing CT and DSA procedures to be performed on the same device, thus simplifying the process and minimizing radiation dose. These combined results confirm the successful development of AEMMs, which significantly enhances DSA, CT, and T_2_-weighted MR imaging capabilities. With real-time imaging monitoring and personalized treatment strategies, AEMMs can predict treatment outcomes and prognosis, making them highly promising for broader applications in medical diagnostics.

### 2.5. In Vitro Cytotoxicity

**Figure 6:**
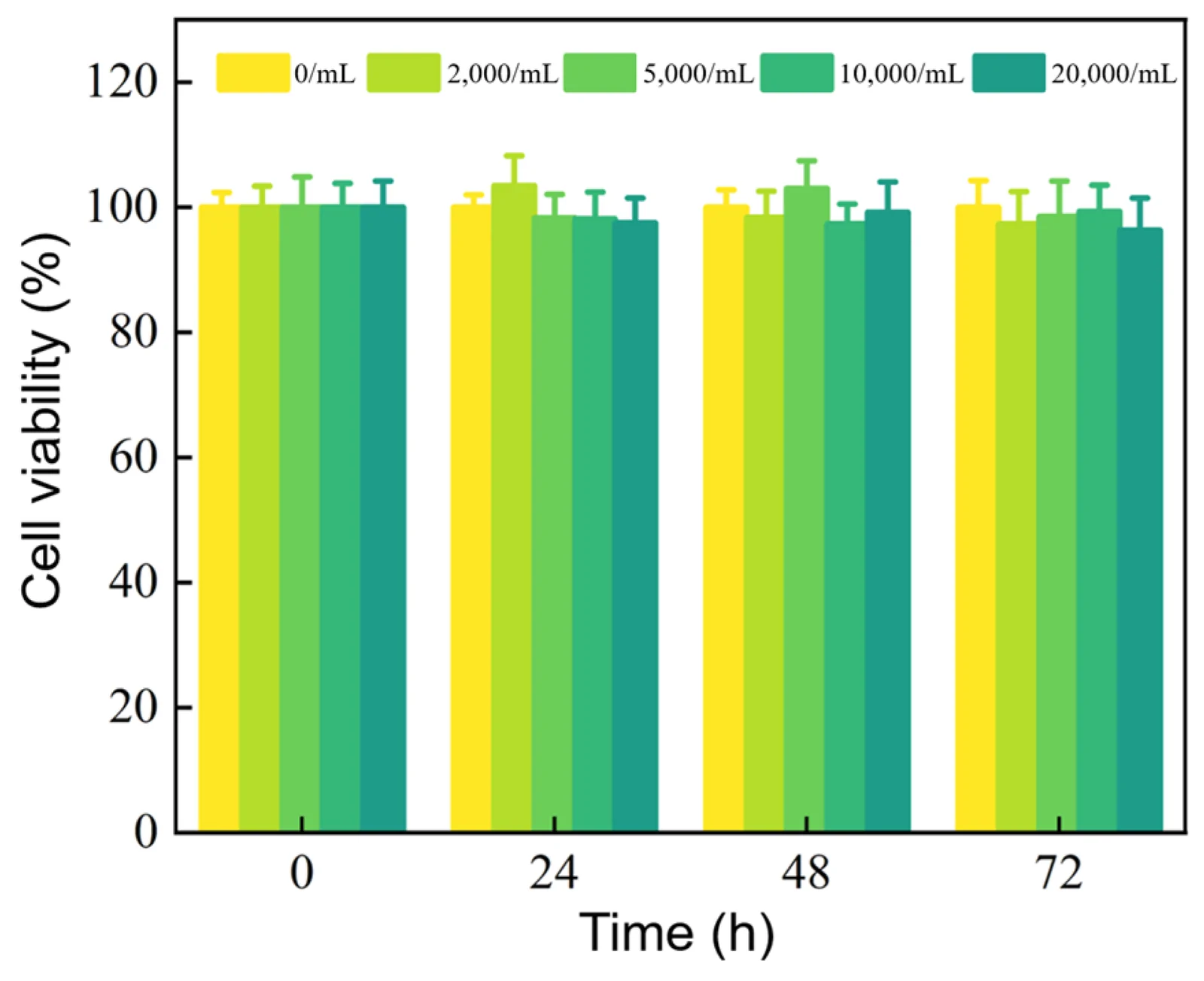
Cell viability of Hep G2 cells incubated with blank AEMMs (0, 2000, 5000, 10,000, or 20,000 microspheres/mL) for 0, 24, 48, and 72 h. Data are shown as mean ± standard deviation (*n* = 6).

For biomedical interventional applications, it is crucial to assess the cytotoxicity of the developed AEMMs. To quantitatively evaluate this, we conducted a CCK-8 assay to measure the cell viability of Hep G2 cells after incubation for 24, 48, and 72 h. The results indicate that even at a concentration as high as 20,000 microspheres/mL, the cytotoxicity of blank AEMMs towards Hep G2 cells is negligible after 72 h of co-incubation (Figure 6).

Furthermore, we evaluated the anticancer activity of drug-loaded AEMMs and determined their IC_50_ values. The IC_50_ (Figure S3) of DOX-loaded AEMMs eluate against HepG2 cells at 48 h was 1.67 μg/mL (equivalent DOX concentration), and that of irinotecan-loaded AEMMs eluate was 11.6 μg/mL (equivalent irinotecan concentration).

## 3. Materials and Methods

### 3.1. Materials

All materials were used as received without further purification. Iron oxide nanoparticles (Fe_3_O_4_ Nps, 50 nm), doxorubicin hydrochloride (DOX, 98%), irinotecan hydrochloride (99%), liquid paraffin, Span 80, poly (sodium-*p*-styrenesulfonate) (PSS) and agarose were procured from Aladdin Chemical Reagent Co., Ltd. (Shanghai, China). Phosphate-Buffered Salin (PBS, 1×) was purchased from Meilun Biotechnology Co., Ltd. (Dalian, China). D-Sorbitol (98%) and D-Lactic acid (90%) were received from Macklin Biochemical Technology Co., Ltd. (Shanghai, China). Hydrogen chloride (HCl) was obtained from Sinopharm Chemical Reagent Co., Ltd. (Shanghai, China). Iohexol was supplied by Yangtze River Pharmaceutical (Group) Co., Ltd. (Taizhou, China). Commercial microspheres used for comparison: CalliSpheres^®^ (sodium acrylate–vinyl alcohol copolymer hydrogel microspheres, 100–300 μm) was obtained from Jiangsu Hengrui Medicine Co., Ltd. (Lianyungang, China); DC Beads^®^ (polyvinyl alcohol hydrogel microspheres, 100–300 μm) was purchased from Biocompatibles UK Limited (Farnham, UK). The cell counting kit-8 assay (CCK-8) was used as purchased from Dojindo Laboratories (Kumamoto, Japan). Ultrapure water (UPW, 18.2 MΩ) was utilized as the solvent for all experiments. The morphology of AEMMs was carefully observed using a Zeiss Sigma 300 scanning electron microscope (SEM, Zeiss, Oberkochen, Germany). Ultraviolet-visible (UV-vis) extinction spectra were collected using Shimadzu UV2600 spectrophotometer (Shimadzu, Shanghai, China).

### 3.2. Synthesis of AEMMs

Dissolve 0.96 g agarose in 20 mL iohexol at 70 °C to prepare a 4.8 wt% aqueous phase, while mechanically stirring at 400 rpm to ensure uniformity. Add 0.2 g Fe_3_O_4_ Nps (50 nm) and 0.5 g PSS to the aqueous phase, mixing thoroughly with stirring and water bath heating to form a homogeneous mixture. Gradually add the aqueous phase to an oil phase composed of 75 mL of paraffin and 3.5 g of Span 80, while stirring at 200 rpm and maintaining the temperature at 70 °C. Continue stirring the reaction mixture at 70 °C for 50 min, then rapidly cool the system to 10 °C. Wash the microspheres several times with 95% ethanol and ultrapure water. Separate microspheres with a particle size of 100–300 μm using standard sieves and store them in ultrapure water. The AEMMs are now ready for use. Particle size distribution was determined by laser diffraction particle size analysis (Mastersizer 3000, Malvern Panalytical, Malvern, UK) and further verified by statistical analysis of optical microscopy and SEM images (*n* = 100 microspheres).

### 3.3. Drug Loading

Due to differences in packaging among brands (e.g., DC beads is available in 2 mL vials, while Callispheres is available in 1 mL or 1 g packages), for convenience in statistical analysis, this study discusses drug loading performance based on 1 mL of microspheres loaded with 100 mg of drug. DC beads and Callispheres were each rinsed three times with ultrapure water before use. The calibration curve for doxorubicin hydrochloride (DOX) was first determined by preparing DOX solutions at concentrations of 20, 40, 60, 80, and 100 µg/mL. For each concentration, 3 mL of solution was taken, and the absorbance at 480 nm was measured using a UV spectrophotometer. Three parallel experiments were conducted to calculate the calibration curve. Next, 1 mL of microspheres was mixed with 4 mL of a 25 mg/mL DOX solution for drug loading, simulating the clinical loading method. The absorbance of the supernatant at 480 nm was measured at 0, 15, 30, 60, 90, and 120 min using a UV spectrophotometer. The amount of DOX was calculated using the calibration curve (y = 0.0173x + 0.0266, R^2^ = 0.9997). Drug loading capacity (mass of drug per mL of packed microspheres) was calculated as follows: Drug loading capacity = Initial DOX amount − DOX amount in supernatant at each time point.

The loading procedure for Irinotecan follows the same steps as described above, 25 mg/mL Irinotecan is dissolved in a mixture of lactic acid and sorbitol (concentrations of 0.0009 g/mL and 0.0225 g/mL, respectively). The pH was adjusted to 3.2–3.6 with 0.2 mol/L sodium hydroxide solution. Lactic acid serves as a solubilizer by protonating irinotecan to improve aqueous solubility, while sorbitol acts as an isotonic stabilizer. The absorbance of the supernatant was measured at 369 nm using an ultraviolet spectrophotometer, and the amount of Irinotecan was calculated by the calibration curve (y = 0.031x + 0.0409, R^2^ = 0.9991). The drug loading is calculated by the following formula: Drug loading capacity = Initial Irinotecan dose − Irinotecan in supernatant at each time point.

### 3.4. Drug Release

Place 0.2 mL of DOX-loaded DC beads, Callispheres, and AEMMs into 50 mL of PBS at pH 5.6 and pH 7.4, respectively, and incubate at 37 °C with shaking to evaluate differences in drug release amounts and release rates. Samples are collected at 15, 30 min, 1, 2, 4, 6, 8, 12, 18, 24, 48, 72, 96, and 120 h, and the solvent is replenished to 50 mL after each collection. The same method is used to evaluate the drug release of the three types of microspheres loaded with Irinotecan, with samples collected at 15, 30 min, 1, 2, 4, 6, 8, 12, 18, 24, and 48 h. The drug concentration is determined by UV spectrophotometry. The release amount at each time point is calculated as follows: Release amount = Cn × V − Cn-1 × Residual volume. Cumulative release amount was defined as the sum of release amounts at all time points, normalized to per mL of packed microspheres.

### 3.5. Photothermal Capability Testing of AEMMs

0.25 mL AEMMs was loaded into the Eppendorf tube, and the 808 nm laser near-infrared laser (Oceanhood, Shanghai, China) was irradiated for 3 min.

The laser spot diameter was approximately 6 mm, and the power density was calibrated to 0.33 W/cm^2^ using an optical power meter (PM100D, Thorlabs, Newton, NJ, USA) before each experiment. An infrared thermal imager (DT-988, Shenzhen Huashengchang Technology Industrial Co., Ltd., Shenzhen, China) was used to capture thermal images every 30 s. To evaluate photothermal stability, AEMMs (0.25 mL) were irradiated with the 808 nm laser (0.33 W/cm^2^) for 3 min, then the laser was turned off and the sample was allowed to cool to room temperature. This irradiation–cooling cycle was repeated five times in total.

### 3.6. In Vitro Multimodal Imaging Characterization of AEMMs

To assess CT Hounsfield unit (HU) values, measurements were conducted using a SOMATOM Force CT system (Siemens Healthineers, Forchheim, Germany) with the parameters set to 120 kV, 150 mA, and a slice thickness of 0.5 mm. Image reconstruction was performed on a Syngo. Via VE 40B workstation (Siemens Healthineers, Forchheim, Germany), and CT values were extracted using the manufacturer’s software. Contrast enhancement was quantified by linear fitting of HU values for each sample. The magnetic resonance relaxivity of AEMMs was evaluated using a 3.0 Tesla MRI scanner (Philips 3.0T Ingenia CX, Philips Medical Systems, Best, The Netherlands). T_2_ measurements were performed using a T_2_ calculation sequence with parameters set as TR = 2000 ms and TE = 10, 20, 30, 40, 50, and 60 ms. All imaging experiments were performed in vitro, not in vivo TACE procedures. The Fe and I concentrations in AEMMs were controlled by adjusting the feeding amounts of Fe_3_O_4_ nanoparticles and iodixanol during synthesis, and quantified by inductively coupled plasma optical emission spectrometry (ICP-OES) and UV-vis spectrophotometry, respectively.

### 3.7. In Vitro Biocompatibility

Human hepatocellular carcinoma HepG2 cells were obtained from Shanghai institute for Biological Sciences (Shanghai, China). Cells were cultured in Dulbecco’s Modified Eagle Medium (DMEM) supplemented with 10% (*v*/*v*) fetal bovine serum (FBS) and 1% (*v*/*v*) penicillin–streptomycin solution, under a humidified atmosphere containing 5% CO_2_ at 37 °C. For the cytotoxicity assessment of blank AEMMs, HepG2 cells were seeded at a density of 5 × 10^3^ cells per well into 96-well plates (*n* = 5 per condition) and allowed to adhere for 24 h. Subsequently, the culture medium was replaced with 100 µL fresh complete growth medium containing either AEMMs (0, 2000, 5000, 10,000, or 20,000 microspheres/mL), followed by incubation for 24, 48, or 72 h. Cells treated with complete growth medium only served as a negative control group. Cell viability was quantified using the CCK-8 assay according to the manufacturer’s protocol. At least three independent experiments were done.

To determine the half-maximal inhibitory concentration (IC_50_) of drug-loaded AEMMs against HepG2 cells, experiments were performed using an indirect dosing approach with microsphere leachate. Briefly, doxorubicin-loaded AEMMs or Irinotecan-loaded AEMMs were incubated in serum-free cell culture medium at 37 °C with constant agitation for 48 h to allow drug release. Subsequently, the supernatant was collected by centrifugation and sterilized by filtration through a 0.22 μm sterile filter to obtain the 48 h drug-loaded leachate. The actual concentrations of doxorubicin and Irinotecan in the leachate were quantified by high-performance liquid chromatography (HPLC), and the leachate was serially diluted based on the measured concentrations to generate six concentration gradients: 0.25, 0.5, 1.0, 2.0, 4.0, and 8.0 μg/mL for doxorubicin-loaded leachate, and 2.0, 4.0, 8.0, 16.0, 32.0, and 64.0 μg/mL for irinotecan-loaded leachate. HepG2 cells were seeded at a density of 5 × 10^3^ cells per well into 96-well plates (*n* = 5 per condition) and allowed to adhere for 24 h. Then cells were treated with the drug-loaded leachate at the aforementioned concentration gradients. A blank cell control group and a blank microsphere leachate control group were set up in parallel. The plates were then incubated in a cell culture incubator for an additional 48 h. Following incubation, cell viability was assessed using the CCK-8 assay. Dose–response curves were fitted by nonlinear regression analysis using GraphPad Prism 8.0 software, the IC_50_ values of the 48 h leachate from drug-loaded AEMMs against HepG2 cells were calculated from the fitted curves.

### 3.8. Statistical Analysis

The data are presented as the mean ± standard deviation. Comparisons between two groups were performed by *t* tests, and paired *t* tests were used for pre–post comparisons of the same group. SPSS Statistics 23.0 and GraphPad Prism 8.0 were used for statistical analysis and plotting. *p* < 0.05 was considered significant difference.

For size and morphological analysis of AEMMs before and after drug loading: (1) Image acquisition: Optical and SEM images were captured with an IX73 inverted bright-field microscope (Olympus, Tokyo, Japan) and a Zeiss Sigma 300 SEM (Zeiss, Germany), respectively. For each group, at least 10 random fields were imaged across triplicate independent sample batches to eliminate selection bias. (2) Number of particles analyzed: A total of 100 intact microspheres per group were randomly selected for size quantification, consistent with the sample size standard in Section 3.2. (3) Image analysis workflow: All dimensional measurements were performed using ImageJ software (version 1.54p, Wayne Rasband, National Institutes of Health, Bethesda, MD, USA). Each image was first calibrated using its embedded scale bar to establish the pixel-to-length conversion factor. The contour of each selected microsphere was then manually traced to derive the equivalent Feret diameter. Finally, raw diameter data were exported to GraphPad Prism 8.0 for statistical calculation of mean diameter and standard deviation.

## 4. Conclusions

In summary, AEMMs are fabricated via a facile one-step emulsion method for combinational TACE therapy of advanced HCC. Compared with conventional embolic agents, the agarose scaffold features excellent biocompatibility, thermal stability and low manufacturing cost while circumventing complex fabrication processes. AEMMs achieve ultrahigh drug loading capacities of 89.87 ± 1.78 mg/mL for DOX and 90.60 ± 0.84 mg/mL for irinotecan, comparable to CalliSpheres^®^ and nearly twofold higher than DC Beads^®^. Moreover, co-encapsulated iodixanol and Fe_3_O_4_ nanoparticles endow AEMMs with integrated CT/DSA/MRI imaging capability for real-time intraoperative guidance and postoperative efficacy evaluation, a feature rarely available in commercial embolic microspheres. Notably, the Fe_3_O_4_-mediated photothermal effect (temperature > 50 °C within 3 min) enables synergistic embolization–chemo–photothermal therapy, addressing the key limitation of insufficient efficacy of single-modality TACE for advanced tumors. Collectively, AEMMs hold strong translational potential as a multifunctional embolic agent for precision interventional HCC therapy, and future work will focus on in vivo efficacy and biosafety evaluation to further advance clinical translation.

## Data Availability

The raw data supporting the conclusions of this article will be made available by the authors on request.

## Supplementary Materials

The following supporting information can be downloaded at: https://www.mdpi.com/article/10.3390/molecules31203626/s1, Table S1: List of Abbreviations. Figure S1: The size distribution of AEMMs before (248.26 ± 5.71 μm) and after (254.38 ± 7.23 μm) DOX loading. Figure S2: Representative SEM image of drug-loaded AEMMs. Figure S3: Dose-response curve of HepG2 cells treated with 48h eluates from drug-loaded AEMMs.
