# Supplementary material for: Agarose-Encapsulated Multifunctional Microspheres with High Drug Payload and Multimodal Imaging Capability for Arterial Embolization–Associated Hyperthermia Therapy
Source: Molecules. 2026 Oct 12;31(20):3626. doi: 10.3390/molecules31203626 (PMC13707394; doi:10.3390/molecules31203626)
Supplement: Supplementary file 1 [file molecules-31-03626-s001.zip › molecules-4545240-supplementary.pdf]

Agarose-Encapsulated Multifunctional Microspheres with High Drug Payload and Multimodal Imaging Capability for Arterial Embolization–Associated Hyperthermia Therapy

Yuqing Zhang [1,*], Shuo Huang [1], Quanming Kou [1], Yindong Xu [2], Yizhou Xu [2], Lei Chen [3], Xiangyue Wang [1] and Changsheng Shi [2,*]

[1] School of Automation, Hangzhou Dianzi University, Hangzhou 310018, China
[2] Department of Interventional Vascular Surgery, The Third Affiliated Hospital of Wenzhou Medical University, Wenzhou 325200, China
[3] Department of Clinical Laboratory, The Third Affiliated Hospital of Wenzhou Medical University, Wenzhou 325200, China
* Correspondence: zhangyuqing@hdu.edu.cn (Y.Z.); shichangsheng@wmu.edu.cn (C.S.)

Table S1. List of Abbreviations.

Abbreviation	Full Name
AEMMs	Agarose-encapsulated multifunctional microspheres
CCK-8	Cell counting kit-8
CT	Computed tomography
MRI	Magnetic resonance imaging
DSA	Digital subtraction angiography
IC_{50}	Half-maximal inhibitory concentration
TACE	Transarterial chemoembolization
UV-vis	Ultraviolet-visible
PSS	Poly(sodium *p*-styrenesulfonate)
HCC	Hepatocellular carcinoma

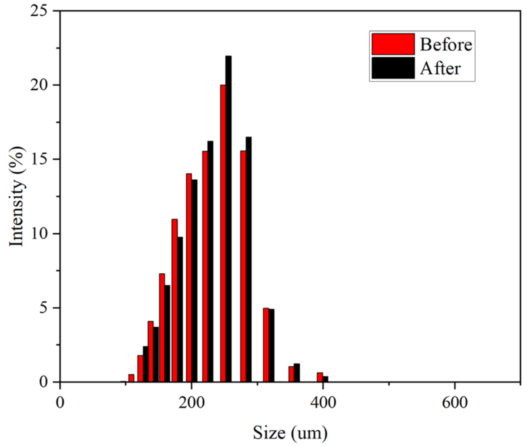

Figure S1. The size distribution of AEMMs before (248.26 ± 5.71 μm) and after (254.38 ± 7.23 μm) DOX loading.

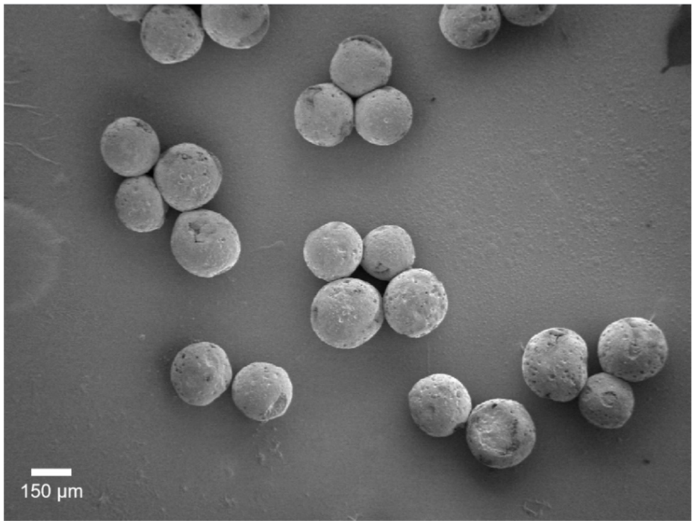

Figure S2. Representative SEM image of drug-loaded AEMMs.

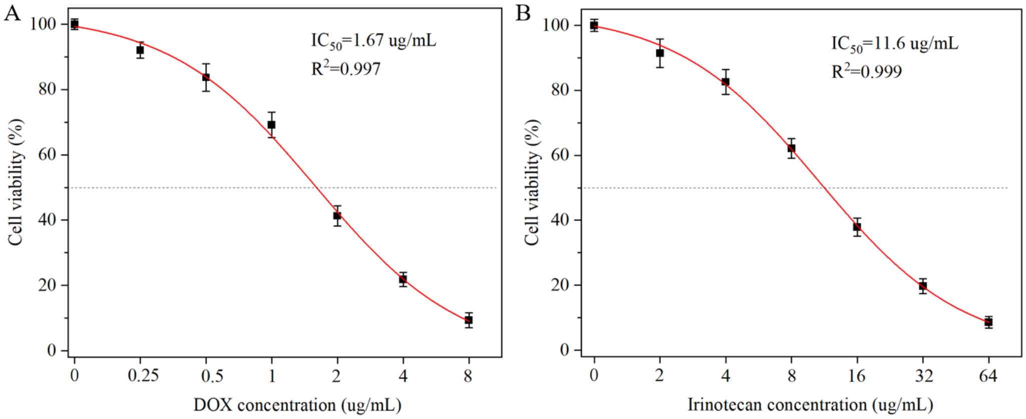

Figure S3. Dose-response curve of HepG2 cells treated with 48h eluates from drug-loaded AEMMs.
